# Reduced Pain Sensation and Reduced BOLD Signal in Parietofrontal Networks during Religious Prayer

**DOI:** 10.3389/fnhum.2017.00337

**Published:** 2017-06-28

**Authors:** Else-Marie Elmholdt, Joshua Skewes, Martin Dietz, Arne Møller, Martin S. Jensen, Andreas Roepstorff, Katja Wiech, Troels S. Jensen

**Affiliations:** ^1^Center of Functionally Integrative Neuroscience, Aarhus University HospitalAarhus, Denmark; ^2^NIDO, Regional Hospital West JutlandHerning, Denmark; ^3^Interacting Minds Centre, Department of Culture and Society, Aarhus UniversityAarhus, Denmark; ^4^Oxford Centre for Functional Magnetic Resonance Imaging of the Brain, University of OxfordOxford, United Kingdom; ^5^Danish Pain Research Center, Aarhus University HospitalAarhus, Denmark

**Keywords:** cognitive pain modulation, expectations, fMRI, naloxone, religion

## Abstract

Previous studies suggest that religious prayer can alter the experience of pain via expectation mechanisms. While brain processes related to other types of top-down modulation of pain have been studied extensively, no research has been conducted on the potential effects of active religious coping. Here, we aimed at investigating the neural mechanisms during pain modulation by prayer and their dependency on the opioidergic system. Twenty-eight devout Protestants performed religious prayer and a secular contrast prayer during painful electrical stimulation in two fMRI sessions. Naloxone or saline was administered prior to scanning. Results show that pain intensity was reduced by 11% and pain unpleasantness by 26% during religious prayer compared to secular prayer. Expectancy predicted large amounts (70–89%) of the variance in pain intensity. Neuroimaging results revealed reduced neural activity during religious prayer in a large parietofrontal network relative to the secular condition. Naloxone had no significant effect on ratings or neural activity. Our results thus indicate that, under these conditions, pain modulation by prayer is not opioid-dependent. Further studies should employ an optimized design to explore whether reduced engagement of the frontoparietal system could indicate that prayer may attenuate pain through a reduction in processing of pain stimulus saliency and prefrontal control rather than through known descending pain inhibitory systems.

## Introduction

Religious prayer is known to alter the experience of pain in some contexts. In a previous study, we have shown that under certain circumstances devout Protestants are able to reduce pain sensation through internal prayer (Jegindø et al., 2013b), an effect that was found to be tightly linked to expectancy. Expectations have also been identified as a key mechanism underlying other types of top-down modulation of pain such as placebo analgesia (Benedetti et al., 2005, 2011b; Benedetti, 2009; Meissner et al., 2011). Since expectation mechanisms have a core role in modulating pain during both placebo and prayer, pain regulation by prayer might be associated to placebo-like effects in terms of neural responses and neurotransmission. While brain processes and neurotransmission related to placebo analgesia have been studied extensively, no research has been conducted on the potential effects of active religious coping. Thus here, we aimed at investigating the neural mechanisms during pain modulation by prayer and the possible involvement of the opioidergic system.

The descending control system involved in pain processing and analgesia includes both opioid and non-opioid dependent systems. The opioid dependent system is known to be particularly powerful. It originates in the mesencephalic periaqueductal gray (PAG), which via a link in the rostral ventromedial part of the medulla (RVM) projects to the dorsal horn of the spinal cord, where it inhibits nociceptive signaling neurons, including those projecting to rostral sites. Generally, placebo-mediated analgesia may recruit the opioid system (Levine et al., 1978; Petrovic et al., 2002; Zubieta et al., 2005; Scott et al., 2007, 2008; Wager et al., 2007; Eippert et al., 2009). However, evidence exists to document non-opioid mechanisms in some types of placebo (Vase et al., 2005; Petrovic et al., 2010; Benedetti et al., 2011a). In addition to more general uncertainties about the underlying neural mechanisms, it is unknown whether pain reduction associated with religious coping strategies is related to the function of the opioid-dependent control system or if it relies on a non-opioid-linked mechanism. The opioid antagonist naloxone has been shown to effectively block both opioid-induced analgesia and opioid-dependent placebo analgesia (Levine et al., 1978; Levine and Gordon, 1984). Naloxone is therefore an excellent tool for investigating the dependence of pain modulation effects, following both active and placebo-related treatments, on endogenous opioid transmission.

In the present study, participants were asked to perform internal prayers to either God or to a non-religious personal stand-in “Mr. Hansen” while pain stimulation was applied in an fMRI paradigm. In one session, participants were administered the opioid antagonist naloxone prior to proceeding. During scanning, participants were asked to rate the intensity, unpleasantness, and expectancies associated with their pain experiences in each condition. Thus we investigated (1) if it was possible to confirm that prayer modulates the experience of pain (indexed by ratings), (2) which brain structures are associated to the effect (fMRI), and (3) if such modulation is mediated by opioidergic or non-opioidergic mechanisms (drug manipulation).

## Materials and Methods

### Participants

Thirty-one healthy, right-handed volunteers participated in the study. Participants were recruited among highly devout Danish Protestants from fractions within the Danish Lutheran Church. All participants reported to attend to religious services regularly, to practice prayer at least daily, and had no history of pain disorders, neurological or psychiatric illness, or daily use of analgesics or medicine. Three participants were excluded prior to the second scanning session (one participant failed to provide ratings for > 25% of the trials in session 1, while two participants with complete data from the first session were excluded prior to the second session because one suspected pregnancy and the other started a medical treatment), resulting in a final sample size of *N* = 28 (F/M: 16/12; mean age: 24.0 years, range: 21–32). The study was approved by the Ethical Committee of Central Region Denmark (20110001), and all procedures including written consent were in accordance with APA guidelines and the Declaration of Helsinki. None of the participants had any religious objections to the study.

### Experimental Design

The study design was a 2 × 2 factorial design with the factors PRAYER (religious vs. non-religious) and DRUG (saline vs. naloxone). Saline and naloxone were administered in separate scanning sessions, separated by 3 weeks. Drug session order was counterbalanced between participants, with information about order withheld from participants and experimenters involved in data collection (double-blinded cross-over design). In each session, there were 30 pain stimulus trials, with participants cued to pray to God in 15 of the trials (Religious Prayer) and to Mr. Hansen in the other 15 (Secular Prayer). Trials from the two prayer conditions were interleaved within each session and the order switched between sessions. Half of the participants started with Religious Prayer followed by Secular Prayer in the first session and vice versa in the second session, and the other half of the participants followed the inverted order. Participants were counterbalanced to condition order by an assistant blinded to the purpose of the study and the identity of the participants. Dependent measures were reports of expected pain intensity prior to each trial, pain intensity and pain unpleasantness following each trial, and BOLD response measured using fMRI.

### Procedure

#### Painful Electrical Stimulation

Painful electrical stimuli were applied by a constant current stimulator (Model DS7A, Digitimer, Hertfordshire, United Kingdom) and four concentric electrodes (WASP, Speciality Developments, Kent, United Kingdom) delivering square waveform pulses of 500 μs duration to the back of the left hand. A random inter-stimulus interval of 300–500 ms with a mean of 400 ms and 50 stimulations for the total stimulation time of 20 s was chosen in order to minimize habituation. Furthermore, the site of stimulation was varied within a 2 ***×*** 2 cm patch between trials. Participants were randomized to stimulus site order. Pre-calibration was performed on the test day on the bed of the scanner immediately before the naloxone administration and the subsequent scanning session. We used a manual staircase procedure and the same trains as during scanning, except only for 100 ms per stimulation. For each stimulation, participants would rate the pain verbally (range 0–100, 0 indicating “no pain sensation” and 100 indicating “most intense pain sensation imaginable”). Levels corresponding to 80 were used for stimulation during the scanning session.

#### Naloxone Administration

Depending on the session, participants were injected with a bolus of 0.15 mg/kg naloxone (Naloxone B Braun, B Braun, Copenhagen, Denmark) known to antagonize endogenous opioid systems (Amanzio et al., 2001; Eippert et al., 2009), or saline via an intravenous line into a left cubital vein immediately before the image acquisition and approximately 5 min before the beginning of the EPI sequence. In both cases, the bolus was diluted in a sterile solution of saline and injected over 90–120 s. Naloxone is usually effective in 1–2 min when administered i.v. It reaches the brain almost instantly as the drug is highly lipophilic. The plasma half-life is 60–90 min. None of the participants had any adverse effects following the two i.v. administrations.

#### Instructions and Debriefing

An instruction session was organized prior to the experiment. Here, participants were told that the purpose of the study was to investigate possible correlations between prayer and pain sensations, with a specific focus on potential correlations between subjective ratings of pain and pain-related brain activity. Participants were not informed of the intention to compare the two prayer conditions. Instead, they were told that the Mr. Hansen task would be used as a neutral control for task-related activity in areas known to be involved in language and memory processes.

Participants were not informed about the full purpose of the naloxone injection prior to the study (this was done during debriefing instead). Participants were informed about naloxone, its pharmacological properties, and possible side effects. They were assured that there were no dangerous side effects known in the current dosage, and they were explicitly told that they would most likely not feel or notice any effects of the injection [consistent with previous research showing no effects of naloxone on mood or experimental pain stimulation (Grevert and Goldstein, 1978)]. Participants were told that the pharmacological properties of naloxone allowed us to visualize relevant pain modulation areas in the brain. Upon completion of the second session, participants were fully debriefed and were informed that naloxone is known to antagonize opioid-dependent pain inhibition.

On the day of the experimental session, the instructions were repeated orally, inclusion criteria were confirmed, and written consent was obtained. Participants were instructed that in the Religious Prayer condition, they should construct and repeat a personal prayer to God to assist in coping with the painful stimulation, and that in the Secular Prayer condition they should do the same, except this time direct the prayer to “Mr. Hansen.” As in Jegindø et al. (2013b), the Secular Prayer condition was used to control for the possible distractive effects of prayer, and participants were asked to construct a prayer as they would otherwise do as if directed to God, only replacing “God” with “Mr. Hansen.” A detailed description and discussion of the tasks is given elsewhere (Jegindø et al., 2013b).

#### Experimental Tasks

Prior to scanning, participants were allowed to test and practice the experimental paradigm outside the scanner and without pain stimulation until they felt comfortable with the instructions and the response method (see below). Specifically, they were trained on how to rate expected pain intensity, experienced pain intensity, and experienced pain unpleasantness using a visual analog scale (VAS). The display for the VAS was anchored at each end as follows: “no pain sensation” and “most intense pain sensation imaginable” for expected and experienced pain intensity ratings and “no unpleasantness” and “most unpleasantness imaginable” for unpleasantness ratings (Price et al., 1994). Ratings were provided by moving a red cursor on the scale with a trackball using the right hand.

**FIGURE 1:**
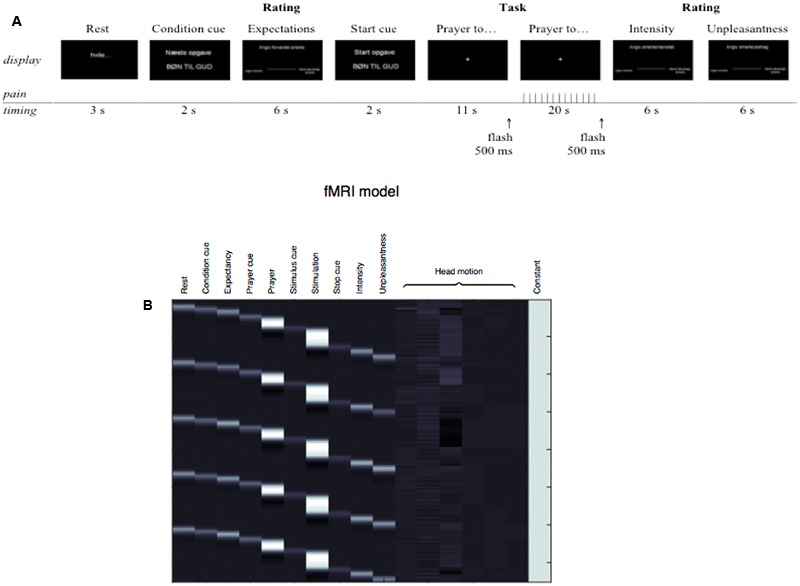
**(A)** Illustration of the paradigm timing. The two conditions were presented 15 times each for each of the two sessions and alternated between trials. After the 10th and the 20th trial in each session there was a 30 s additional rest phase (black display). During scanning, a trial began with a 3 s period in which the word “rest” was presented on the screen. After the rest display, a condition cue informed participants of the next task (i.e., prayer to God or Mr. Hansen). Expectations were rated immediately before the task. Participants were instructed to close their eyes and begin the task (pray to God or Mr. Hansen) when presented with the “Start task: PRAYER TO GOD/MR. HANSEN” start cue display and until cued to stop. The first 11 s of this interval was stimulus free, during which time no painful stimulation as applied. This was included to facilitate mood/concentration on the subsequent task. After 11 s, the stimulus display flashed white for 500 ms, which was perceived by participants even though they had their eyes closed, to indicate the onset of the noxious stimulation and to avoid startle and movement artifacts. Painful stimulus was then applied for 20 s. These periods of prayer+stimulation were modeled as contrast of interests in the fMRI-data analyses. After this, a 500 ms white flash indicated that participants should stop praying and open their eyes. They were then asked to rate the intensity and then the unpleasantness of the painful stimulation. Six seconds were given for each of the M-VAS ratings. **(B)** Model describing the experiment.

During scanning, a trial began with a 3 s period in which the word “rest” was presented on the screen and participants were not given any specific instructions except to look at the display. This was to allow time to recover from the previous pain stimulation and to prepare for the next. Following this, a cue was presented for 2 s, to instruct participants who to pray to (prayer condition) during the upcoming pain stimulation. Next, participants were asked to use the VAS scale to rate their expected pain for the next stimulus, with 6 s allowed to make the rating. When presented with a start cue, participants were then instructed to close their eyes and begin praying until cued to stop. The first 11 s of this interval was stimulus free, during which time no painful stimulation as applied. This was included to facilitate mood/concentration on the subsequent task. After 11 s, the stimulus display flashed white for 500 ms, which was perceived by participants even though they had their eyes closed, to indicate the onset of the noxious stimulation and to avoid startle and movement artifacts. Painful stimulus was then applied for 20 s. After this, a 500 ms white flash indicated that participants should stop praying and open their eyes. They were then asked to rate the intensity and then the unpleasantness of the painful stimulation (see **Figure 1A**). Six seconds were given for each of these ratings. Presentation^®^ software (Neurobehavioral Systems Ink., Albany, CA, United States) was used for stimulus presentation, to trigger the pain stimulator, and record the ratings provided.

#### Validation of Religiosity, Task Authenticity, and Blinding

A questionnaire on religious belief and use of prayer was given to all participants to verify that participants in fact firmly believed in God, practiced prayer frequently, and believed in the support of God. In order to assess the vividness of the religious experience during the experiment, a post-test questionnaire was used in which participants were asked to rate contact to and presence of God/Hansen and the perceived authenticity of the prayers to God/Hansen using a 10-point Likert scale. The same questionnaires have been used in a previous experimental study on prayer and pain modulation (see details in Jegindø et al., 2013b) and similar questionnaires have been used in fMRI studies on prayer (Schjødt et al., 2008, 2009). Finally, in order to validate the blinding, participants were asked to indicate their suspected treatment order by indicating in which session they believed they received the naloxone bolus.

#### fMRI Image Acquisition and Preprocessing

T2^∗^-weighted, gradient-echo echo-planar images (EPI) were acquired on a 3 Tesla Siemens Magnetom Trio Scanner (Siemens, Erlangen, Germany), equipped with a 32-channel head coil. Each volume consisted of 37 slices of 3 mm thickness acquired in an interleaved acquisition order with the following parameters: TR = 2000 ms, TE = 30 ms, flip angle = 90°, with an in-plane resolution of 3 × 3 × 3 mm and FOV = 192 × 192 mm. Soft cushions were used to minimize head movement.

Image pre-processing and statistical analysis was performed using Statistical Parametric Mapping (SPM8, revision 4667). The functional images of each participant were realigned (Friston et al., 1995), spatially normalized to MNI space using the SPM8 EPI template and trilinear interpolation (Ashburner and Friston, 1999) and smoothed with a 8 mm FWHM isotropic kernel. The time-series in each voxel was high-pass filtered at 128 s to remove slow drift.

### Analyses

#### fMRI Analysis

Statistical analysis of the fMRI data was performed using a two-level general linear model (Worsley and Friston, 1995). A model encoding the experimental paradigm and subject responses was fitted to each subject’s BOLD time-series, including regressors for each of the two 2 × 2 experimental conditions ‘Religious Prayer, saline,’ ‘Secular Prayer, saline,’ ‘Religious Prayer, naloxone,’ and ‘Secular Prayer, naloxone.’ This first-level model also included the affine motion parameters estimated during realignment to remove the effects of head movement (**Figure 1B**). Inference at the group level was performed using summary statistics in a one-sample *t*-test. This included a test per main effect of CONDITION: [(‘Religious Prayer, saline’ + ‘Religious Prayer, naloxone’) – (‘Secular Prayer, saline’ + ‘Secular Prayer, naloxone’)] and [(‘Secular Prayer, saline’ + ‘Secular Prayer, naloxone’) – (‘Religious Prayer, saline’ + ‘Religious Prayer, naloxone’)] and DRUG: [(‘Religious Prayer, saline’ + ‘Secular Prayer, saline’) – (‘Religious Prayer, naloxone’ + ‘Secular Prayer, naloxone’)] and [(‘Religious Prayer, naloxone’ + ‘Secular Prayer, naloxone’) – (‘Religious Prayer, saline’ + ‘Secular Prayer, saline’)]), as well as their interaction: [(‘Religious Prayer’ – ‘Secular Prayer’)_saline_ – (‘Religious Prayer’ – ‘Secular Prayer’)_naloxone_] and [(‘Religious Prayer’ – ‘Secular Prayer’)_naloxone_ – (‘Religious Prayer’ – ‘Secular Prayer’)_saline_]. Finally, we tested for a linear relationship between behavioral ratings and BOLD response at the group level. These included differences in VAS ratings of pain intensity, pain unpleasantness, and expected pain intensity between the Religious and Secular conditions as a predictor of the BOLD response in the Religious compared to Secular contrast. Statistical parametric maps were corrected for multiple comparisons using Gaussian random field theory with a cluster-level family-wise error (FWE) rate at *p* < 0.05.

#### Behavioral Analysis

In order to assess the relative authenticity of the experimental conditions, Wilcoxon signed-rank test was used to compare post-questionnaire scores for prayers to God and prayers to Mr. Hansen. A paired *t*-test was used to test whether stimulation intensities were different in the two sessions. Pearson’s chi-square was used to test whether participants could accurately guess the session order (i.e., test if the blinding was effective). In order to evaluate the behavioral modulation of pain and expectancy of pain from prayer, separate 2 (DRUG) × 2 (PRAYER) repeated measures ANOVAs were performed on pain intensity, pain unpleasantness, and expected pain intensity ratings. To investigate whether expectations contributed to the perception of pain, within each condition and within each session, mean pain expectancy ratings were regressed against mean pain intensity ratings. To further explore the influence of expectations on effects of PRAYER condition, we also regressed changes in mean expectancy ratings between the prayer and secular trials, against changes in mean intensity ratings between the two sorts of trials, separately for each drug session.

## Results

### Validation

The pre-questionnaire confirmed that participants had a high belief in God (*M* = 9.46, *SD* = 0.79), practiced prayer frequently (weekly *M* = 15.75, *SD* = 9.59), and had a strong belief in God’s support (*M* = 8.75, *SD* = 1.51).

**Table 1** shows the comparison of the post-questionnaire scores between the religious and the non-religious condition. Results confirm the relative authenticity of the religious condition as participants scored high on contact to and presence of God and on the “naturalness” of the prayers to God. Conversely, the scores on these parameters for Mr. Hansen were very low and significantly different from scores related to God, indicating that participants did not experience contact to or presence of Mr. Hansen as a metaphysical entity. There was no difference on any of these measures between sessions (all *ps* > 0.05). Importantly, there was no difference in stimulation intensity between sessions (see **Table 1**), and Pearson’s chi-squared test confirmed that participants could not accurately guess their treatment order (*p* = 0.490), which indicates that the blinding was effective.

**Table 1:** Comparison of post-questionnaire ratings (between conditions) and stimulation intensity (between sessions).

Variable	*M*	*SD*	*Z*^a^*/T^b^*	*p*	*r*
Perceived contact (0–10)			-4.55^a^	<0.001	-0.86
Religious prayer	7.91	1.63			
Secular prayer	1.23	1.62			
Perceived presence (0–10)			-4.63^a^	<0.001	-0.87
Religious prayer	6.86	1.94			
Secular prayer	0.93	1.26			
Perceived naturalness of the prayers (0–10)			-4.63^a^	<0.001	-0.87
Religious prayer	7.54	2.33			
Secular prayer	1.38	1.59			
Average stimulus levels (mA)			1.11^b^	0.287	0.21
Saline	16.39	10.57			
Naloxone	14.27	7.43			

*The table shows the results of comparisons. ^a^Wilcoxon signed-rank test; ^b^paired *t*-test.*

### Ratings

**FIGURE 2:**
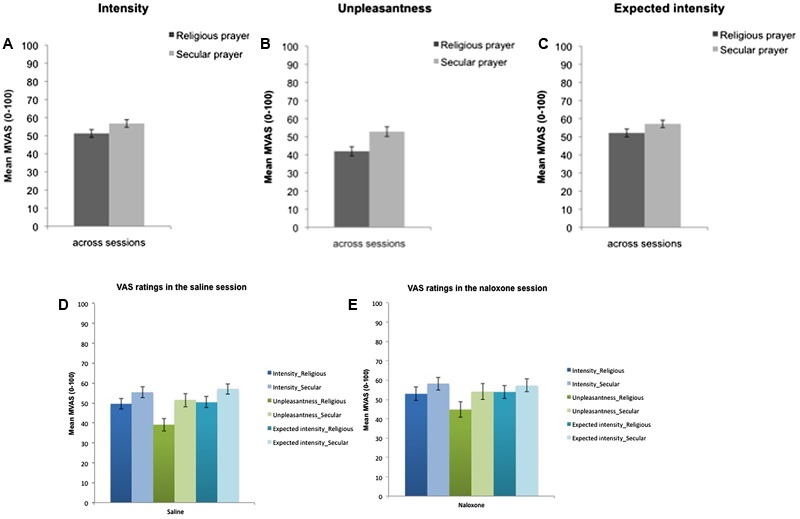
Mean ratings of **(A)** pain intensity, **(B)** pain unpleasantness, and **(C)** expected pain intensity across naloxone and saline sessions and mean ratings of these variables separately in saline **(D)** and naloxone **(E)** sessions. Error bars indicate mean standard error.

The variances in subjective ratings of pain intensity, pain unpleasantness, and expected pain intensity across sessions are illustrated in **Figures 2A–E**. The ANOVAs revealed a significant main effect of condition for pain intensity [*F*(1,27) = 11.73, *p* = 0.002, $\eta_{p}^{2}$ = 0.30], pain unpleasantness [*F*(1,27) = 28.03, *p* < 0.001, $\eta_{p}^{2}$ = 0.51], and expected pain intensity [*F*(1,27) = 7.15, *p* = 0.013, $\eta_{p}^{2}$ = 0.21]. We found no main effects of drug and no condition × drug interactions (the lowest *p*-value was main effect of drug on unpleasantness: *p* = 0.141). These results show that the participants experienced a significant reduction in pain intensity and pain unpleasantness and expected less pain intensity in the Religious Prayer condition compared to the Secular Prayer condition for both treatments, and it indicates that this effect was not opioid-dependent. Across sessions, the perceived pain intensity was reduced by 11% and pain unpleasantness by 26% during religious prayer (see also **Figures 2A,B,D,E**).

Results of the regression analyses on pain intensity are summarized in **Table 2**. In the Religious Prayer condition expectancy predicted 78% of the variance in the pain intensity ratings for the saline session and 86% of the variance in these ratings in the naloxone session. In the Secular Prayer condition, expectations accounted for 70% of the variance in pain intensity scores in the saline session and 89% of the variance in these ratings in the naloxone session.

**Table 2:** Prediction of pain intensity.

Model	*R^2^*	*F*	*p*	β
Religious prayer_saline_	0.777	87.10	<0.001	
Expectancy				0.881
Religious prayer_naloxone_	0.859	152.71	<0.001	
Expectancy				0.927
Secular prayer_saline_	0.695	59.13	<0.001	
Expectancy				0.833
Secular prayer_naloxone_	0.890	203.18	<0.001	
Expectancy				0.944

*Results of the regression analyses on expected pain intensity levels.*

Results of the regression analysis on changes in pain intensity across prayer conditions revealed a significant effect of pain expectancy in both the Saline (β = 0.904, *t* = 16.803, *p* < 0.001) [*R*^2^ = 0.839, *F*(1,54) = 282.3, *p* < 0.001] and the Naloxone conditions (β = 0.915, *t* = 15.144, *p* < 0.001) [*R*^2^ = 0.809, *F*(1,54) = 229.3, *p* < 0.001].

### Neuroimaging Results

**FIGURE 3:**
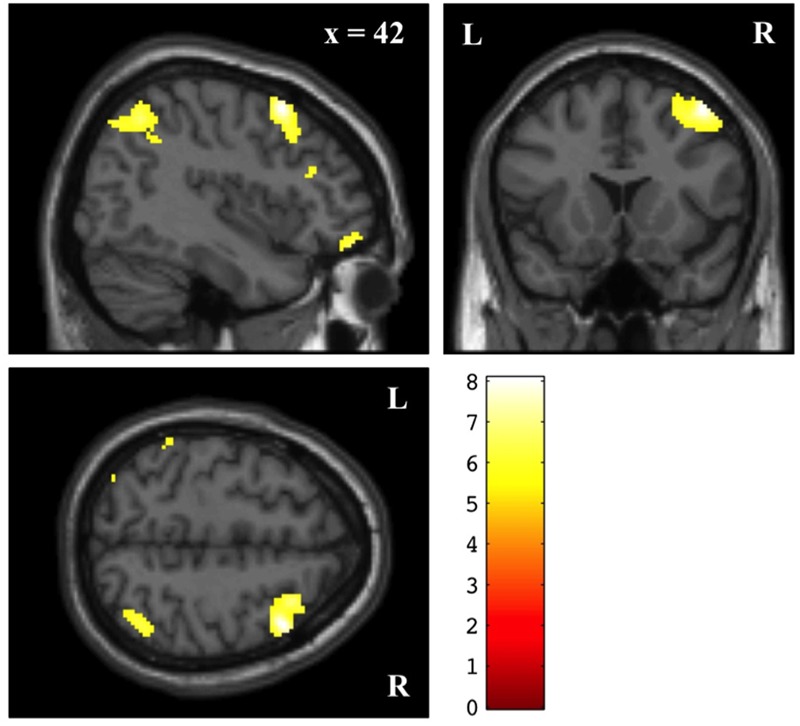
Main effect of prayer (Secular Prayer > Religious Prayer) across naloxone and saline sessions. Voxel-wise statistical parametric maps (*p*FWE < 0.05) superimposed on SPM canonical anatomical image. See **Table 4** for a complete list of foci.

Across sessions and conditions, we observed pain-related brain activation in the contralateral insula and other relevant regions during painful stimulation (see **Table 3**) (Peyron et al., 2000; Apkarian et al., 2005; Duerden and Albanese, 2013). Across sessions we found a significant BOLD increase in Secular Prayer relative to Religious Prayer in a network of parietofrontal regions including the right frontal eye field, DLPFC, and LOFC, precuneus, and bilateral posterior parietal cortex (PPC) at *p*FWE < 0.05 (**Figure 3** and **Table 4**). No significant relative increase was found for the reverse contrast (i.e., Religious Prayer > Secular Prayer). Also there was no significant main effect of drug or interaction between condition and drug for the Religious Prayer and the Secular Prayer conditions (either way). We found no significant association between the VAS ratings of pain intensity, unpleasantness, and expected pain intensity and BOLD responses.

**Table 3:** Brain regions displaying significant BOLD activation during painful stimulation across conditions and sessions.

Putative anatomical region	Brodmann area	Peak MNI			Voxels *k*	*T*	*p*FWE
		*x*	*y*	*z*			
R Anterior insula		42	16	-14	16571	15.00	<0.001
L Inferior parietal cortex	40	-52	-58	50	554	9.71	<0.001
R Medial frontal gyrus	8	4	26	48	1833	8.98	<0.001
L Middle frontal gyrus	6/8	-48	10	50	300	7.95	<0.001
R Inferior occipital gyrus	18/19	34	-94	-10	86	7.15	0.002
L Inferior occipital gyrus	18/19	-32	-94	-14	117	6.93	0.003
L Middle temporal gyrus	21	-64	-42	-6	124	6.79	0.003
R Middle temporal gyrus	21	64	-28	-18	24	6.57	0.005
L Culmen		-14	-38	-24	77	6.39	0.008
L Orbitofrontal cortex	10/11	-46	48	-14	25	6.17	0.013
L Putamen		-26	-2	-6	20	5.97	0.020
L Superior frontal gyrus	9	-24	56	34	10	5.82	0.027
L Post-central gyrus	40	-64	-22	18	4	5.74	0.032
L Cerebellum		-22	-62	-24	6	5.61	0.042
L Putamen		-28	-2	6	2	5.61	0.043
L Superior frontal gyrus	8	-18	32	58	2	5.55	0.048

*Cluster size *k* = number of contiguous voxels for that cluster. *P*-values corrected for multiple comparisons, *p*FWE < 0.05. L, Left; R, Right.*

**Table 4:** Brain regions displaying significant BOLD activations for Secular Prayer compared to Religious Prayer across sessions.

Putative anatomical region	Brodmann area	Peak MNI			Voxels *k*	*T*	*p*FWE
		*x*	*y*	*z*			
R Middle frontal gyrus	8	42	16	54	564	8.05	<0.001
R Inferior parietal cortex	40	42	-58	50	379	6.69	0.003
R Dorsolateral prefrontal cortex	46	50	34	24	157	6.61	0.004
R Orbitofrontal cortex	11	42	50	-16	44	6.37	0.007
L Inferior parietal cortex	40	-38	-54	42	22	6.09	0.012
L Inferior parietal cortex	40	-52	-46	56	12	5.92	0.018
R Precuneus		10	-72	44	4	5.55	0.039
L Superior parietal lobe		-34	-72	54	2	5.69	0.029

*Cluster size *k* = number of contiguous voxels for that cluster. *P*-values corrected for multiple comparisons, *p*FWE < 0.05. L, Left; R, Right.*

## Discussion

The aim of the present study was to investigate possible pain modulating effects of religious prayer as an example of active religious coping. Using fMRI and a double-blind naloxone/saline cross-over design, we explored associated bran mechanisms and the possible involvement of opioidergic transmission. Results show that prayer decreases pain sensation for devout Protestants in agreement with previous findings (Jegindø et al., 2013b). Across sessions pain intensity was reduced by 11% and pain unpleasantness by 26% during the religious condition. Expectancy accounted for the majority of the variance and the change in pain intensity, supporting the notion that expectation mechanisms are a key factor in pain sensation reduction by prayer (Jegindø et al., 2013b). In addition, pain reduction was not affected by naloxone in the current study, suggesting that the effect is probably dependent on a non-opioid-linked system. The fMRI results show influence of neither opioidergic nor non-opioid pain inhibitory brain mechanisms, but demonstrate a relative decrease in BOLD activity in a network of parietofrontal regions for the religious condition compared to the secular contrast condition. This is consistent with recent findings of non-opioidergic cognitive pain modulation and the notion of multiple pathways in pain control independent of descending inhibitory mechanisms (Vase et al., 2005; Petrovic et al., 2010; Wager et al., 2011). Zeidan and colleagues recently reported a similar finding where a naloxone infusion failed to reverse meditation-induced analgesia. They found no significant differences in pain intensity or pain unpleasantness reductions between the meditation+naloxone and the meditation+saline groups (Zeidan et al., 2016). Our finding also implies, however, that the pain reducing effect of prayer, in this particular setting, is not associated with an “activation” of specific cortical regions.

This study confirmed that, at least in the current setting, devout Protestants were able to reduce pain sensation through prayer. Post-scan ratings of contact to God, presence of God, and perceived authenticity of the prayers were relatively high for the Religious Prayer condition, but very low during Secular Prayer (see Results). In agreement with Schjødt et al. (2008, 2009) and Jegindø et al. (2013b), this indicates that participants were able to perform prayers in the scanner which were comparable with prayers in a more natural setting. It also shows, however, that praying to Mr. Hansen was perceived as a rather unnatural task that did not involve contact to or presence of a Mr. Hansen. Post-scan reports further indicated that participants in general experienced that the Religious Prayer condition helped them to disconnect from part of the painful input. In addition, many participants expressed that they had to concentrate more when constructing prayers to Mr. Hansen, but that it did not distract them from the pain. It is possible that these issues may have influenced the results (see below). None of the participants, however, expressed any religious concerns to either of the experimental tasks.

Surprisingly, no brain regions were more “active” during Religious Prayer compared to Secular Prayer. Furthermore, we found no association between behavioral measures of pain reduction and the difference in BOLD signal. As a consequence, it remains unknown which brain mechanisms are involved in regulating pain in this context, and the reason for the decrease of activity during Religious Prayer compared to Secular Prayer is not clear. One possibility is that saliency detection/monitoring systems and attentional processing of the painful input is reduced during prayer to God vs. prayer to Mr. Hansen, as participants become less attentive and less sensitive to the noxious input (Grant and Rainville, 2009; Legrain et al., 2009, 2011; Iannetti and Mouraux, 2010). Participants were all devout Protestants who practiced prayer frequently. As indicated by the post-scan reports, it is possible that in the Religious Prayer vs. Secular Prayer condition, participants were able to rely on an internal “script” (and possibly pre-established neural pathways) for constructing the prayer, whereas the Secular Prayer condition resulted in a relative increase in working memory, executive control, and cognitive appraisal as indicated by the relative increase in the PPC, precuneus, frontal eye field, DLPFC, and LOFC. Increases in BOLD and rCBF have previously been shown in these areas during pain stimulus detection/perception (Peyron et al., 2000; Apkarian et al., 2005). In addition, the PPC, precuneus, frontal eye field, DLPFC, and LOFC have also been reported as belonging to functional attentional networks (Corbetta et al., 1991; Pardo et al., 1991; Fredrikson et al., 1995; McCarthy et al., 1997; Nobre et al., 1997; Peyron et al., 1999). In agreement with previous studies of attentional aspects of pain processing, our data might indicate that a large cortical network is recruited during Secular Prayer > Religious Prayer, which is associated with attentional-executive activity related to the noxious stimulus (Peyron et al., 2000; Casey et al., 2001). This corresponds to the relative increase in parietofrontal regions during Secular Prayer, and, in this perspective, the right laterality of these activations is consistent with previous findings of right lateralisation during attentional modulation, especially in PPC and DLPFC (Pardo et al., 1991; Corbetta et al., 1993; Gitelman et al., 1996; Peyron et al., 1999).

While we only have experimental evidence from one previous study on neuronal pain modulation associated with religious beliefs (Wiech et al., 2009), a number of studies have investigated the role of meditation on pain modulation (Grant and Rainville, 2009; Buhle and Wager, 2010; Grant et al., 2010, 2011; Perlman et al., 2010; Gard et al., 2011). Recently, Gard et al. (2011) have shown that mindfulness practitioners are able to reduce pain unpleasantness by 22%, and that this effect is associated with a relative decrease in the lateral prefrontal cortex and increase in the right posterior insula and the rACC during anticipation of pain. In line with the study by Gard and colleagues we found a relative decrease in BOLD in lateral prefrontal regions during prayer and a 26% reduction in pain unpleasantness. It may be the case that mindfulness meditation and prayer share a mechanism involving prefrontal executive control and affective properties of pain experience that helps to reduce pain as participants dissociate from part of the negative elements of the pain rather than attempting to control the bottom-up experience (see also Jegindø et al., 2013a). Similarly, in the context of Christian intercessory prayer, Schjødt and colleagues demonstrated frontoparietal BOLD decreases in response to prayers believed to have healing properties (Schjødt et al., 2011).

### Limitations and Future Directions

Several limitations of this study should be acknowledged. First, the lack of a ‘no-pain’ or a ‘non-painful stimulation’ control condition in the current design renders difficult the interpretation of brain responses related to pain processing and regulation. As a consequence of this compromise, the current design is suited to investigating the interaction between religious prayer and opioid functioning, but lacks a sensory control, which could have made possible more direct analyses of pain processing mechanisms involved. In addition, the absence of a control condition involving non-painful stimulation renders possible that the so-called pain-related activation might reflect somatosensory perception independent from pain. We leave these more direct investigations for future research. A major challenge of this research field is, to ensure relative authenticity of the task, while adapting to the research method at hand. In particular, this trade-off relates to timing issues and the requirement of several repetitions of, e.g., ‘prayer on demand.’ A favorable alternative to the current task could be to use a much simpler practice, which does not require the same cognitive effort and mood induction as personal prayer. Based on previous studies by Schjødt et al. (2008, 2009), it would be interesting to test whether, e.g., the Lord’s Prayer might modulate pain experience and engage cognitive pain inhibitory mechanisms.

Our behavioral and neuroimaging results indicate that pain modulation through religious prayer does not seem to be opioid-sensitive. The lack of signal increase in areas known to be involved in descending pain regulatory brain processes may also relate to inter-individual differences in the ability to engage these systems. In order to address this issue, future studies may wish to include a large group of predefined high-responders. Alternatively, it may be favorable to explore other potential routes of endogenous pain regulation, such as the endocannabinoid system (Benedetti et al., 2011a) or dopaminergic reward and motivational systems of the dorsal striatum (Scott et al., 2007, 2008; Schjødt et al., 2008), using an approach similar to ours.

A promising venue of research would be to further explore the role of expectations in pain regulation from cultural beliefs and practices by, for example, actively inducing conditioned expectations of analgesia (manipulate stimulus intensity), enhancing the effect pharmacologically [e.g., morphine conditioning (see Amanzio and Benedetti, 1999)], or by employing open vs. hidden administration of painkillers (or antagonists) in order to efficiently study the potential placebo-like effects of prayer. Finally, since we do not see a correlation between changes in pain ratings and BOLD in the current study it is difficult to rule out issues related to compliance and report biases.

In summary, the current study presents pain reduction by prayer linked to expectation mechanisms and we find significant decreases in BOLD in attentional and executive systems during religious vs. secular prayer. This mechanism seems to rely on non-opioidergic systems. We suggest that, in contrast to current knowledge of descending pain inhibition, prayer might attenuate pain through a reduction in processing of pain stimulus saliency and prefrontal control. Thus, as a religious coping strategy, prayer may in some circumstances allow devout participants to cope with pain by dissociating from part of the negative input of the stimulus and hence decrease the demand for selecting the appropriate response. Because pain reduction is not directly associated to an activation of specific brain areas in the context of this study, the results only hints at a “true” analgesic effect. Further research is warranted in order to clarify the neural mechanisms of pain control by cultural beliefs and practices, and our findings provide good leads for following this up in future studies. Importantly, it would be important to include both proper sensory and task-related control conditions to further test the hypothesis, that prayer is associated with dissociation from bottom-up sensory processing.

## Author Contributions

E-ME, JS, KW, AR and TJ: design and conceptual idea behind the study. E-ME, JS, AM, MJ: responsible for setup and acquisitions. E-ME, JS and MD: analyses. E-ME and JS: preparation of the manuscript draft, tables and figures. All authors have revised the work critically and provided their final approval for publication in Frontiers in Human Neuroscience, hereby agreeing to be accountable for all aspects of the work.

## Conflict of Interest Statement

The authors declare that the research was conducted in the absence of any commercial or financial relationships that could be construed as a potential conflict of interest.
